# A New Dentinal Obtundent

**Published:** 1899-03

**Authors:** 


ARTICLE VIII.

### A NEW DENTINAL OBTUNDENT.

A new candidate for favor as a dentinal anesthntic is just now undergoing the experimental stage, with promise thus far of a high degree of efficiency. The preparation is a product of the chemical laboratory of McKesson and Robbins, 91 Fulton street, New York, and is termed vapocaine. It consists of a fifteen per cent. solution of cocain hydrochlorid in ethyl ether.

The production of such a solution is by no means the simple problem which it might at first glance appear to be, as under orainary circumstances cocain hydrochlorid is practically insoluble in ether. The efficiency of cocain as a dentinal anesthetic has heretofore been unavailable for several reasons, all of which are the result of the lack of a satisfactory method for securing the penetration of cocain salts into the dentinal stiucture. Aqueous solutions of cacain salts fail to penetrate the dentin, because the difference in density between them and the fluids of the dentinal tubuli is too slight to create the condition for rapid diffusion. The ethereal solution of the cocain alkaloid is deficient in anesthetic property. The production of an ethereal solution of a cocain salt secures a preparation possessing not only anesthetic properties, but also the difference in density relative to the fluids in the dentinal tubuli necessary for rapid diffusion.

The action of vapocain as explained by the manufacturers is as follows:

They have found that if the end of a capillary glass tube be placed in contact with a pledget of cotton, saturated with water, covered with anilin, the anilin solution will rise by capillary attraction to a considerable height in the tube. If now a pledget of cotton, saturated with vapocain, be applied to the opposite end of the glass tube and the whole arrangement be gently heated over a water bath, the ether solution carrying with it a certain amount of the cocain salt rapidly penetrates the tube, driving the anilin solution out and displacing it. In a short time, however, evaporation of the ether takes place, leaving a deposit of the cocain salt as an incrustation on the inner walls of the capillary glass tube throughout the entire length, after which, the diffusive factor of the ether having been eliminated by the evaporation, the anilin solution is again drawn into the tube by capillary attraction, dissolving in its passage the incrustation of the cocain salt from the walls of the tube.

It is believed that the result of the application of vapocain to the open ends of the dentinal tubuli in a carious cavity is analogous to that described in the foregoing experiment with the glass tube, with the further result that the solution of the cocain salt deposited in the dentinal tubuli by the natural fluids of the tooth exerts the well-known physiological anesthetizing effect of cocain on the sensory elements of the dentinal fibrillæ. A limited number of tests in the clinic in the operative department have yielded remarkably satisfactory results on hypersensitive dentin. The subject is one of very great importance; and as the investigation of this interesting preparation will be continued until its value or otherwise has been fully established, we hope to present more definite data and some conclusions in regard to it in a future issue of the journal.—*Pacific Medico-Dental Gazette.*

